# Changes in Fluoroquinolone-Resistant *Streptococcus pneumonia* after 7-Valent Conjugate Vaccination, Spain

**DOI:** 10.3201/eid1506.080684

**Published:** 2009-06

**Authors:** Adela G. de la Campa, Carmen Ardanuy, Luz Balsalobre, Emilio Pérez-Trallero, Jose M. Marimón, Asunción Fenoll, Josefina Liñares

**Affiliations:** Instituto de Salud Carlos III, Madrid, Spain (A.G. de la Campa, L. Balsalobre, A. Fenoll); Ciber Enfermedades Respiratorias, Mallorca, Spain (A.G. de la Campa, C. Ardanuy, L. Balsalobre, E. Pérez-Trallero, J. M. Marimón, J. Liñares); Hospital Universitary de Bellvitge, Barcelona, Spain (C. Ardanuy, J. Liñares); Hospital Donostia, San Sebastian, Spain (E. Pérez-Trallero, J.M. Marimón)

**Keywords:** Streptococcus pneumoniae, streptococci, DNA topoisomerases, fluoroquinolones, ciprofloxacin, antimicrobial resistance, MLST, PFGE, Spain, research

## Abstract

Among 4,215 *Streptococcus pneumoniae* isolates obtained in Spain during 2006, 98 (2.3%) were ciprofloxacin resistant (3.6% from adults and 0.14% from children). In comparison with findings from a 2002 study, global resistance remained stable. Low-level resistance (30 isolates with MIC 4–8 μg/mL) was caused by a reserpine-sensitive efflux phenotype (n = 4) or single topoisomerase IV (*parC* [n = 24] or *parE* [n = 1]) changes. One isolate did not show reserpine-sensitive efflux or mutations. High-level resistance (68 isolates with MIC ≥16 μg/mL) was caused by changes in gyrase (*gyrA*) and *parC* or *parE*. New changes in *parC* (S80P) and *gyrA* (S81V, E85G) were shown to be involved in resistance by genetic transformation. Although 49 genotypes were observed, clones Spain^9V^-ST156 and Sweden^15A^-ST63 accounted for 34.7% of drug-resistant isolates. In comparison with findings from the 2002 study, clones Spain^14^-ST17, Spain^23F^-ST81, and ST88^19F^ decreased and 4 new genotypes (ST97^10A^, ST570^16^, ST433^22^, and ST717^33^) appeared in 2006.

## Abstract 2

Four new genotypes appeared in 2006 after childhood vaccination was begun.

The bacterium *Streptococcus pneumoniae* is a serious cause of illness and death and a major etiologic agent of community-acquired pneumonia, meningitis, and acute otitis media. Pneumococcal resistance to antimicrobial drugs (including β-lactams, macrolides, tetracycline, and cotrimoxazole) has become a worldwide problem (*1*); new fluoroquinolones are being used as therapeutic alternatives for treatment of adult patients with community-acquired pneumonia (*2*). Resistance to fluoroquinolones in *S*. *pneumoniae* can be acquired by point mutations, intraspecific recombination (*3*) or interspecific recombination with the *S*. *mitis* group (*3**–**7*). Resistance is caused mainly by amino acid changes in quinolone resistance–determining regions (QRDRs) of the subunits of DNA topoisomerase IV (topo IV; parC_2_ and parE_2_) and DNA gyrase (gyrA_2_ and gyrB_2_) enzymes that control DNA topology. In addition, fluoroquinolone efflux also contributes to resistance (*8*). Genetic and biochemical studies have shown that for most fluoroquinolones, such as ciprofloxacin and levofloxacin, topo IV and gyrase are primary and secondary targets, respectively (*9**–**13*). However, gyrase is the primary target for moxifloxacin (*14*).

Although current prevalence of fluoroquinolone resistance in pneumococci is <5% (*15**–**17*), surveillance is necessary. Introduction of the 7-valent conjugate pneumococcal vaccine (PCV7), which includes serotypes such as 6B, 9V, 14, and 23F that are often associated with resistance to fluoroquinolones and other antimicrobial drugs, has resulted in changes in the epidemiology of invasive pneumococcal disease (*18**–**20*). Since the introduction of PCV7 in Spain in late 2001, ≈47% of children have been vaccinated (*21*).

In this study, we investigated the prevalence of fluoroquinolone-resistant pneumococci in Spain during 2006. Mutations in the QRDRs of *parC*, *parE*, and *gyrA* were identified, and the presence of reserpine-sensitive fluoroquinolone efflux was determined. In addition, resistance associations with other antimicrobial drugs and characteristics of drug-resistant clones were determined. To better evaluate changes in the epidemiology of resistance after the introduction of PCV7 in children, we compared our results with those of a similar study that tested isolates from 2002.

## Methods

### Bacterial Isolates, Serotyping, Susceptibility Testing, and Genetic Transformation

We studied 4,215 *S*. *pneumoniae* isolates from 2 hospitals (in Barcelona and San Sebastián), and a sample from 110 hospitals throughout Spain (Spanish Reference Laboratory, Madrid). Of the isolates, 2,682 were from adults, 1,400 from children, and 133 from persons whose ages were unknown. A total of 2,101 (49.9%) isolates were obtained from blood or other sterile sites; 1,055 (25%) from the lower respiratory tract; 960 (22.8%) from the upper respiratory tract, otic and conjunctival sites; and 99 (2.3%) from other sites. Isolates were confirmed as *S*. *pneumoniae* by standard methods, and serotypes were determined by the Quellung reaction. Ciprofloxacin susceptibility was determined by broth microdilution tests (Sensititer; Trek Diagnostics Inc., East Grinstead, UK) and by agar dilution according to the Clinical and Laboratory Standards Institute guidelines (*22*). Reserpine-sensitive fluoroquinolone efflux phenotype was determined as described (*23*). We performed genetic transformation as described (*24*) by using *S*. *pneumoniae* strains R6 and T1 (*25*) as receptors. For selection of transformants, we used media plates containing 1 μg/mL (R6 derivatives) or 8 μg/mL (T1 derivatives) of ciprofloxacin.

### Pulsed-Field Gel Electrophoresis and Multilocus Sequence Typing

Pulsed-field gel electrophoresis (PFGE) patterns were determined by using *Sma*I and *Apa*I as described (*24*) and compared with 26 representative clones of the Pneumococcal Molecular Epidemiology Network (*26*). Isolates with patterns varying by <3 bands were considered to represent the same PFGE type (*27*). Multilocus sequence typing was performed as described (*28*) with representative isolates of PFGE types shared by >3 isolates (www.mlst.net). We analyzed selected strains representative of dominant clones from the 2002 study by multilocus sequence typing.

### PCR Amplification and DNA Sequence Determination

Oligonucleotides parE398 (*29*) and parC152 (*10*) were used to amplify *parE* and *parC* QRDRs. All isolates yielded fragments of 1.6 kb, with the exception of ciprofloxacin-resistant (CipR) isolates CipR17, CipR39, CipR74, and CipR76, which yielded fragments of ≈5, 5, 5, and 7 kb, respectively. These PCR fragments were sequenced as described (*24*). Oligonucleotides gyrA44 and gyrA170 (*29*) were used to amplify and sequence *gyrA* QRDRs. Oligonucleotides antUP and antDOWN (*4*) were used to detect the *ant* gene.

## Results

Among the 4,215 isolates studied, 98 were CipR. Of these isolates, 30 (30.6%) showed low-level resistance (LL-CipR, MICs 4–8 μg/mL) and 68 (69.4%) high-level resistance (HL-CipR, MICs 16–128 μg/mL) (Table 1). By age group, the prevalence of CipR was 0.14% (2/1,400) among isolates from pediatric patients (<15 years of age) and 3.6% (96/2,682) among isolates from adult patients. Resistance was higher among noninvasive pneumococci (3.3%, 70/2,114) than among invasive isolates (1.3%, 28/2,101, p<0.001). The highest rate of Cip resistance was found for isolates from adults >64 years of age (Table 1). All HL-CipR isolates were from adult patients; most (53/68, 77.9%) were isolated from sputum. CipR isolates showed high rates of resistance to antimicrobial drugs. However, these rates were lower than those found in the 2002 study (Table 1).

**Table 1:** Comparison of 2 surveillance studies on ciprofloxacin-resistant *Streptococcus pneumoniae* isolates in Spain, 2002 and 2006*

Characteristic	No. ciproflaxin resistant/no. isolates (%)		p value
	2002	2006	
Ciproflaxin resistance			
Global	75/2,882 (2.6)	98/4,215 (2.3)	NS
Low-level (MICs 4–8 μg/mL)	14/75 (18.7)	30/98 (30.6)	NS
High-level (MICs >16 μg/mL)	61/75 (81.3)	68/98 (69.4)	NS
In persons <15 years of age	0/978 (0)	2/1,446 (0.14)	NS
In persons 15–64 years of age	22/1,166 (1.9)	34/1,455 (2.3)	NS
In persons >64 years of age	53/738 (7.2)	62/1,314 (4.7)	0.02
PCV7 serotypes	49/75 (65.3)	35/98 (35.7)	<0.001
Other antimicrobial drug resistance	No. resistant/no. ciproflaxin-resistant isolates (%)		
Penicillin MIC >0.12 μg/mL	55/75 (73.3)	44/98 (44.9)	<0.001
Erythromycin MIC >0.5 μg/mL	53/75 (70.7)	53/98 (54.1)	0.03
Clindamycin MIC >1 μg/mL	47/75 (62.7)	45/98 (45.9)	0.03
Chloramphenicol MIC >8 μg/mL	33/75 (44.0)	11/98 (11.2)	<0.001
Tetracycline MIC >4 μg/mL	52/75 (69.3)	39/98 (39.8)	<0.001
Cotrimoxazole MIC >4/76 μg/mL†	51/75 (68.0)	47/98 (47.8)	0.008
Multidrug resistance (>3 drugs)	55/75 (73.3)	48/98 (49.0)	<0.001

*NS, not significant; PCV7, 7-valent conjugate pneumococcal vaccine. Ciproflaxin resistance is defined by Chen et al. (*30*) as an MIC >4 μg/mL. †MIC is 4 μg/mL for trimethoprim and 76 μg/mL for sulfamethoxazole.

The *parC*, *parE*, and *gyrA* QRDRs of the 98 CipR isolates were characterized. Most CipR isolates (93/98) showed low nucleotide sequence variations (<1%) in their QRDRs, but 5 isolates showed high variations (>4%). Four of them were in *parC*, *parE*, and *gyrA*, and only 1 was in *gyrA*. These results suggest an interspecific recombinant origin for these genes. In accordance, all isolates with recombinant *parE* and *parC* genes carried the *ant* gene, typical of the *S*. *mitis* group (*4*), as shown by PCR amplification.

Twenty-one of the 98 isolates had efflux for Cip; 3 of them also had efflux for levofloxacin (Tables 2, 3), and none had efflux for moxifloxacin. Efflux was equally distributed among LL-CipR and HL-CipR isolates. The contribution of the efflux mechanism to resistance in those isolates is unclear. Mutations not previously described that produced changes in parC (D78N, S80P, D83E), parE (I476F), and gyrA (G79A, S81V, E85G, V101I) were found in 8 isolates. To test the contribution of these changes to resistance, transformation experiments using strains R6 or T1 (as R6, parC S79F) as receptors of *parC* or *gyrA* QRDRs, respectively, were performed. The QRDRs of several independent transformants were sequenced to confirm the presence of the same mutation in the donor DNA and MICs of these transformants were determined. Although no transformation was achieved with PCR products carrying parC D78N or parE I476F, transformation to increased resistance was observed with products carrying parC S80P, gyrA S81V, and gyrA E85G changes (Table 2).

**Table 2:** Fluoroquinolone MICs of 30 low-level resistant *Streptococcus pneumoniae* isolates and 5 laboratory strains and amino acid changes in their DNA topoisomerase IV and gyrase genes, Spain, 2006*

No. isolates	Amino acid substitution													Efflux phenotype†
	*parC*				*parE*			*gyrA*			MIC, μg/mL			
	S79	S80	D83		D435	E474		S81	E85		CIP	LVX	MXF	
1	–	–	–		–	–		–	–		4	1	0.12	None
3	–	–	–		–	–		–	–		4–8	2	0.5	CIP
1	–‡	–‡	–‡		–‡	–‡		–‡	–‡		8	4	0.5	CIP
9	F	–	–		–	–		–	–		4–8	1–2	0.25–0.50	None
3	F	–	–		–	–		–	–		4–8	2	0.12–0.25	CIP
1	F	–	–		–	–		–‡	–‡		8	1	0.12	None
1	F‡	–‡*	–‡		–‡	–‡		–‡	–‡		8	2	0.12	CIP, LVX
5	Y	–	–		–	–		–	–		4–8	2	0.12–0.25	None
1	Y	–	–		–	–		–	–		4	2	0.25	CIP
1	–	–	N		–	–		–	–		16	4	0.5	None
1	–	–	N		–	–		–	–		4	2	0.12	CIP
1	–	–	Y		–	–		–	–		4	1	0.5	None
1	–	–	Y		–	–		–	–		8	2	0.5	CIP
1	–	–	–		N	–		–	–		8	2	0.12	None
Laboratory strains§														
R6											0.5	0.25	0.12	None
R6^CS80P^	–	P	–		–	–		–	–		2	1	0.25	None
T1	F	–	–		–	–		–	–		4	2	0.12	None
T1^AS81V^	F	–	–		–	–		V	–		32	32	4	None
T1^AE85G^	F	–	–		–	–		–	G		32	8	2	None

*par, topoisomerase gene; *gyr*, gyrase gene; CIP, ciprofloxacin; LVX, levofloxacin; MXF, moxifloxacin. Only changes involved in resistance are shown. –, no change. Additional amino acid changes not involved in resistance were parC D78N (1 isolate), parC K137 N (9), parC N91D (2 with mosaic *parC* genes), parE I460V (17), parE I476F (1), gyrA S114G (2 with mosaic *gyrA* genes), and gyrA N150H (1 with a mosaic *gyrA* gene). †An isolate was considered to have an efflux phenotype for the indicated fluoroquinolone when a >2-fold decrease in its MIC in the presence of reserpine was observed. ‡Indicates that the residue is located in a recombinant gene. §R6^CS80P^, R6 derivative carrying parC S80P; T1^AS81V^, T1-derivative carrying gyrA S81V; T1^AE85G^, T1-derivative carrying gyrA E85K.

**Table 3:** Fluoroquinolone MICs of 68 high-level resistant *Streptococcus pneumoniae* isolates and amino acid changes in their DNA topoisomerase IV and gyrase genes, Spain, 2006*

No. isolates	Amino acid substitution													Efflux phenotype†
	*parC*				*parE*			*gyrA*			MIC, μg/mL			
	S79	S80	D83		D435	E474		S81	E85		CIP	LVX	MXF	
4	F	–	–		–	–		F	–		64	16–32	4	CIP
21	F	–	–		–	–		F	–		32–128	16–32	2–8	None
1	F	–	–		–	–		L	–		64	32	2	None
1	F	–	–		–	–		V	–		64	32	4	CIP
3	F	–	–		–	–		Y	–		64–128	16–32	4	None
1	F	–	–		–	–		–	G		32	16	4	None
2	F	–	–		–	–		–	K		32–64	16–32	2–4	None
1	Y‡	–‡	–‡		–‡	–‡		F‡	–‡		64	32	4	None
8	Y	–	–		–	–		F	–		32–64	16–32	2–4	None
1	Y	–	–		–	–		F	–		64	32	4	CIP, LVX
1	Y	–	–		–	–		Y	–		64	32	4	None
1	Y	–	–		–	–		–	K		32	16	2	None
1	–	P	–		–	–		F	–		16	4	0.5	None
1	–	–	H		–	–		F	–		32	16	2	CIP
1	–	–	Y		–	–		F	–		32	16	2	CIP
2	–	–	Y		–	–		F	–		32	8–16	2–4	None
1	–	–	N		–	–		–	K		16	8	2	None
3	–	–	–		N	–		F	–		16	8	0.5–2	None
1	–‡	–‡	–‡		N‡	–‡		F‡	–‡		16	4	0.5	CIP
1	F	–	G		–	–		F	–		64	32	4	CIP, LVX
2	F	–	G		–	–		F	–		32–64	32	4	None
1	F	–	G		–	–		L	–		64	64	16	None
1	F	–	H		–	–		F	–		64	32	4	None
2	F	–	N		–	–		F	–		32–64	16–32	4	None
2	F	–	–		N	–		F	–		64–128	32–128	4–32	None
1	F	–	–		N	–		–	K		16	32	4	None
1	F	–	–		–	K		F	–		64	32	4	None
1	F	–	–		–	–		F	A		64	16	4	None
1	F	–	–		–	–		F	K		32	32	4	None

*par, topoisomerase gene; *gyr*, gyrase gene; CIP, ciprofloxacin; LVX, levofloxacin; MXF, moxifloxacin. Only changes involved in resistance are shown. –, no change. Additional amino acid changes not involved in resistance were parC D83E (1), parC K137 N (24), parC N91D (2 with mosaic *parC* genes), parE I460V (47), and gyrA S114G (2 with mosaic *gyrA* genes). †An isolate was considered to have an efflux phenotype for the indicated fluoroquinolone when a >2-fold decrease in its MIC in the presence of reserpine was observed. ‡Indicates that the residue is located in a recombinant gene.

Three of these changes were accompanied by other changes known to be involved in resistance: gyrA G79A with S81F; parC D83E with S79F, and gyrA V101I with S81F. Among 5 T1 transformants obtained with a gyrA QRDR carrying G79A and S81F, 4 carried G79A and S81F and only 1 carried S81F. Because all transformants had identical Cip MICs, results suggest that G79A is not involved in drug resistance. We could not discern the role of parC D83E and gyrA S81F in resistance, given that all R6-transformants had parC D83E and S79F and all T1 transformants had gyrA V101I and S81F. However, given the contribution to resistance of the accompanied mutations, their role in resistance is unlikely.

The contribution of classical and new mutations to Cip resistance described here enabled us to classify resistant isolates (Tables 2, 3). Five LL-CipR isolates did not show changes involved in resistance in their *parC*, *parE*, or *gyrA* QRDRs, including 1 with recombinant genes (Table 2). Four of them showed a reserpine-sensitive efflux phenotype for Cip (Table 2) as a single mechanism of resistance. Among the remaining 25 LL-CipR isolates, 24 had mutations producing changes at parC, and 1 isolate had a single change at parE. Among 68 HL-CipR isolates, 55 (80.9%) had double changes (51 in parC and gyrA and 4 in parE and gyrA), and 13 (19.1%) had triple mutations (7 had 2 changes in parC and 1 change in gyrA; 4 had 1 change in parC, 1 change in parE, and 1 change in gyrA; 2 had 1 change in parC and 2 changes in gyrA). According to Clinical and Laboratory Standards Institute guidelines (*22*), only 3 of the 30 LL-CipR isolates showed intermediate resistance to levofloxacin (MIC 4 μg/mL), and the remaining 27 isolates were susceptible to levofloxacin; all were susceptible to moxifloxacin. HL-CipR isolates showed resistance (n = 66) or intermediate resistance (n = 2) to levofloxacin. Five HL-CipR isolates were susceptible to moxifloxacin, 11 showed intermediate resistance, and 52 were resistant.

Serotype and genotype distributions of CipR isolates of 2002 (*24*) and 2006 were compared (Figure). Although isolates from 2006 belonged to 29 different serotypes, 5 serotypes (14, 9V, 8, 19A, and 6B) accounted for 44.9% of the total. The rate of PCV7 serotypes among CipR isolates decreased (p<0.001) in 2006 (Table 1) because of a decrease in serotypes 23F, 19F, and 6B (Figure, panel A). Forty-nine genotypes were observed among the 98 CipR isolates (Figure, panel B). Clones Spain^9V^-ST156 (21 isolates) and Sweden^15A^-ST63 (13 isolates) accounted for 34.7% of the CipR isolates. Capsular switch events were frequent in these clones (Figure): Spain^9V^-ST156 (12 switches) and Sweden^15A^-ST63 (11 switches). Four new genotypes related to non-PCV7 serotypes, (ST97^10A^, ST570^16^, ST433^22^, ST717^33^, each represented by 3 isolates) emerged in 2006 (Figure, panel B).

**Figure:**
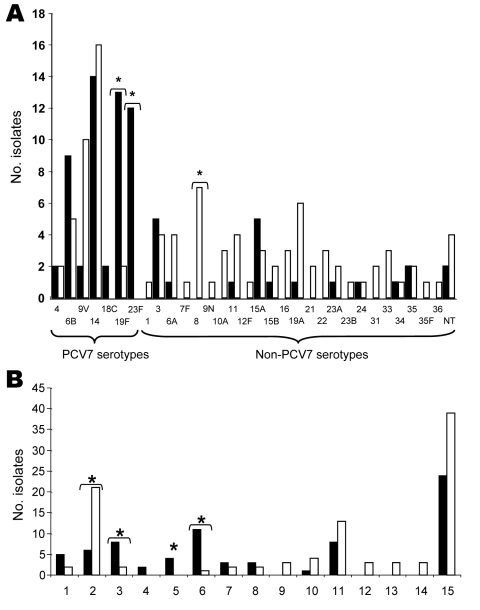
Serotype (A) and genotype (B) distributions of ciprofloxacin-resistant pneumococci isolated in Spain, 2002 and 2006. A total of 75 isolates from 2002 (black columns) and 98 from 2006 (white columns) were compared. Asterisks indicate significant differences (p<0.05) between the 2 years. PCV7, 7-valent conjugate pneumococcal vaccine. Baseline numbers in B indicate various genotypes. 1, Spain^6B^-ST90; 2, Spain^9V^-ST156; 3, Spain^14^-ST17; 4, Netherlands^18C^-ST113; 5, ST88^19F^; 6, Spain^23F^-ST81; 7, Netherlands^3^-ST180; 8, ST260^3^; 9, ST97^10A^; 10, ST62^11A^; 11, Sweden^15A^-ST63; 12, ST570^16^; 13, TS433^22^; 14, ST717^33^; 15, other.

As we observed, isolates that shared the same PFGE pattern also shared identical polymorphisms on their DNA topoisomerase QRDRs. All but 1 of the isolates belonging to the Spain^9V^-ST156 clone had identical polymorphisms, the same found in the ATCC 700671 strain representative of this clone (*15*); the only exception was an isolate with *parC*, *parE*, and *gyrA* recombinant genes.

## Discussion

We observed a stabilization during 2002–2006 in the rates of fluoroquinolone resistance in Spain. Although the rate of Cip resistance in 2002 was 2.6% (2.2% for levofloxacin), it was 2.3% (1.7% for levofloxacin) in 2006. The rates of Cip resistance were also similar for the different age groups (3.5% for adults and 0.14% for children in 2006). However, a decrease in the rate of resistance in persons >64 years of age was found in 2006. Higher levels of resistance were found in *S*. *pneumoniae* isolated from sputa and in isolates from people >64 years of age, who more frequently have chronic obstructive pulmonary disease and who have been treated with multiple regimens of antimicrobial drugs. In accordance, development of fluoroquinolone resistance has been reported for these patients (*31**–**33*). The frequency of HL-CipR resistance in adults was 2.5% (68/2,769), slightly higher than that reported for persons in other countries in Europe (*34*).

Four factors may have contributed to the observed stabilization of resistance rates. These factors are fluoroquinolone use, change in circulating clones, no recommendation of fluoroquinolones for children, and fitness cost of resistance mutations.

A direct correlation between use of fluoroquinolone and prevalence of resistance in *S*. *pneumoniae* has been described (*30**,**35*). Cip use in Spain has remained stable since 1997 at 1.1 defined daily doses (DDDs)/1,000 inhabitants-days, whereas that of levofloxacin and moxifloxacin increased during 2002–2006 (from 0.2 to 0.4 DDDs/1,000 inhabitants-days for levofloxacin and from 0.3 to 0.4 DDDs/1,000 inhabitants-days for moxifloxacin, Agencia Española de Medicamentos, Madrid, Spain; http//agemed.es). Because the borderline activity of Cip against *S*. *pneumoniae* favors acquisition of first-step *parC* mutations (*15**,**36*), we expected that the greater activity of levofloxacin and moxifloxacin would not favor the appearance of resistance, even if one considered their increased use.

Regarding circulating pneumococcal clones, the rate of PCV7 serotypes among CipR isolates decreased from 65.3% in 2002 to 35.7% in 2006 (p<0.001). The same finding was found among CipR isolates from adults >64 years of age (7.2% in 2002 to 4.7% in 2006; p<0.02) and was probably caused by decreased transmission of pneumococci from vaccinated children to adults (*37*). Consequently, we have observed a decrease in 4 multidrug-resistant clones (Spain^23F^-ST81, Spain^6B^-ST90, Spain^14^-ST17, and ST88^19F^) related to PCV7-serotypes. In addition, new clones (ST62^11^, ST97^10A^, ST570^16^, ST433^22^, and ST717^33^) related to non-PCV7 serotypes emerged in 2006. These changes are consistent with those observed among invasive pneumococci after the introduction of PCV7 in Spain in June 2001 (*38*). At present, 2 clones, Spain^9V^-3-ST156 and Sweden^15A^-ST63, could be considered as the major contributors to Cip resistance in Spain, accounting for 34.7% of CipR strains.

Fluoroquinolones are not recommended for children, who are the major reservoir of pneumococci. If fluoroquinolones are given to children, according to recent reports of their safety for such use (*39*), increased prevalence of resistance might occur.

Regarding fitness cost of CipR mutations in *S*. *pneumoniae*, CipR isolates were divided into 3 groups. The first group is composed of 5 isolates without QRDR resistance mutations. Four isolates had a reserpine efflux phenotype. The fifth isolate may have had a different efflux inhibitor or an unknown resistance mechanism. The second group is composed of 25 LL-CipR isolates with single changes at topo IV, whose distribution, 24 at parC and 1 at parE (D435N), is consistent with the low-fitness cost of parC changes (*25*) and the high-fitness cost of the parE D435N change (*40*). The third group is composed of 68 HL-CipR isolates with gyrA changes associated with topo IV changes. GyrA changes mainly occurred at S81 (62/68), whereas changes at E85 were rare (8/68) because of the high-fitness cost of E85 changes (*25*).

The frequency of CipR recombinants in 2006 remained low (5.1%, 5/98 CipR isolates), similar to that in 2002 (6.7%) and that reported previously (*3**,**4*). Four isolates with mosaic *parE-parC* genes and long intergenic regions (4–6 kb) containing the *ant* gene probably originated by recombination with the *S*. *mitis* group (*4*). One of them belongs to the Spain^9V^-ST156 clone and was not typeable. The predominance of this clone and the fact that the recombinant *parE-ant-parC* structure did not impose a fitness cost (*25*) suggest recombinants could become more prevalent in the future.
